# Proactive Community Occupational Therapy Service for Social Participation Development of Thai Adults with Depression: A Grounded Theory Study from Occupational Therapists' Perspective

**DOI:** 10.1155/2021/6695052

**Published:** 2021-07-13

**Authors:** Supaluck Phadsri, Rieko Shioji, Atsuko Tanimura, Sopida Apichai, Jeerawit Jaknissai

**Affiliations:** ^1^Department of Occupational Therapy, Tokyo Metropolitan University, Tokyo 116-8551, Japan; ^2^Department of Occupational Therapy, Chiang Mai University, Chiang Mai 50200, Thailand; ^3^Occupational Therapy Unit, Thanyarak Khon Kaen Hospital, Khon Kaen 40000, Thailand

## Abstract

**Introduction:**

Depression in adulthood decreases social participation in the workplace, family, and community, which further results in decreased work performance and cessation and social isolation. There is a high statistic of outpatient consultation and readmission of Thais with depression, yet the mental health support for remission in community life and social participation remains limited and unclear. Further, due to the lack of mental health professional resources, particularly occupational therapists, there is much to be known regarding how such therapists work to support the development of social participation in Thai adults with depression.

**Objective:**

This research was aimed at understanding the process of how occupational therapists work to redevelop the social participation of community-dwelling Thai adults with depression.

**Method:**

The grounded theory methodology was used in this study. Data were collected through interviews and nonparticipatory observations of 14 participants who had experience providing mental health care in community services. The constant comparative analysis method was employed.

**Result:**

Three concepts illustrated a proactive community occupational therapy service for depression (PCOTS-D), namely, integrating depression care in community occupational therapy service (COTS), supporting meaningful participation, and forming collaborative networks. The PCOTS-D supported the reconnection of social participation by leading from proactive depression care service to COTS and then working to support meaningful participation toward the patient's self-management and building collaborative networks with inter- and intraprofessional teams simultaneously.

**Conclusion:**

The PCOTS-D presented a holistic view of working with community-dwelling Thai adults with depression by considering the importance of the community and researchers' network to redevelop social participation, promote health and recovery, build teams in depression care, and encourage research evidence to enhance the supportive advocacy policy for Thai people with depression.

## 1. Introduction

Depression poses a silent threat to health [1], causes work problems [2, 3], and results in poor relationships with other people [3, 4]. In Thailand, depression is associated with an increased risk of suicide [5]. There is a high prevalence of depression among working-age adults [6]. Furthermore, the number of Thai people readmitted to hospital with depression is almost 10 times greater than initial admissions [6]. Thus, being depressed poses problems to living in the community. Interventions for depression require the integration of biopsychosocial recovery models [7], whereas the management of depression in Thailand is limited to access to medical treatment and psychotherapy [5]. Both medical treatment and psychotherapy are safe, highly tolerable treatment options to enhance the remission of persons with depression [8], but a knowledge gap exists in terms of the translation of such treatments to functional outcomes, particularly social participation.

In the Thai perspective, social participation concerning health and recovery is rarely discussed, and issues of social and health inequalities among Thais continue to persist [9]. In the Thai context, many individuals keep depression a secret and avoid social participation, resulting in isolation and relapse [10]. Hence, in this study, social participation is highlighted as a dimension of mental health recovery [11] and human occupation, consisting of individuals' interactions with their social context [12, 13]. Social participation intervention helps reduce social isolation [11], loneliness [14], and recurrent depressive symptoms [15], whereas the intervention promotes occupational participation in the meaning of life, responsibility, hope, and self-identity [16]. Furthermore, social participation intervention can improve, regain, and maintain socialization during the acute, continuation, and maintenance phases of recovery for individuals with mental illness [8]. However, social participation intervention within the community mental health service is complex, as other problems of individuals with depression, such as loss of interest or pleasure in life activities and impairment in social participation and occupation, should also be tackled [8, 17]. Successful social participation intervention for patients with depression requires both the patients' involvement and community involvement to recreate therapeutic relationships and rehabilitation by recognizing the socioeconomic context, social attitude, and stigma [11, 18, 19]. Thus, an occupational therapist is a crucial healthcare provider that helps individuals with depression engage in social participation optimally in everyday life while living in their community [20], employing the community occupational therapy service (COTS) approach.

Occupational therapy (OT) in Thailand originally began with the vocational rehabilitation in the institutes (Somdet Chaopraya Institute of Psychiatry and Siriraj Hospital) provided by training staff members before an OT school was established in 1980 [21]. Further, COTS was started in the year 2003 by occupational therapists in a community outreach service venture for people with psychiatric issues [22, 23]. Thereafter, COTS was solely developed in a rehabilitation setting, a part of a community hospital located in a community, Chiang Mai province, for people with cerebrovascular disease [24]. The occupational therapists in community hospitals provide COTS associated with the health care and hospital policies that mainly concern a group of individuals with physical disabilities, elders, and children with developmental problems. Conversely, those with mental illnesses in the community fall under the responsibility of outreach services in the psychiatric hospital. Different workplace policies and multilevel of health care delivery system in Thailand (tertiary care: regional hospital and university hospital, secondary care: provincial and/or general hospitals, and primary care: community hospitals and health-promoting hospitals) [25] influence the formation of different specialty areas of occupational therapy (mental health or COTS), providing COTS through community outreach and home visits [23, 24]. Thus, following this trend, Thai occupational therapists attempt to deliver community-based services to reduce readmission rates and bridge the gap in caring for people with mental illness, including depression.

However, there are few occupational therapists [26] and the OT profession is not well known [27]. The service system for depression care in Thailand is transitioning toward community mental health service as per the national plan [27]; however, currently, there is a dearth of evidence-based guidelines to improve social participation for adults with depression, sensitive to the Asian or Thai context. Thus, our research addressed the concepts and processes that occupational therapists use to redevelop social participation among Thai adults with depression. This research was aimed at understanding the process of occupational therapists in redeveloping social participation of community-dwelling Thai adults with depression.

## 2. Context

Chiang Mai province in northern Thailand comprises 25 amphoe (districts), 204 subdistricts, and 2,066 villages with 24 public hospitals (one provincial hospital, two general hospitals, and 21 community hospitals). Amphoe Muang falls under the responsibility of the provincial hospital, which acts as the community hospital for people living in the area. In Chiang Mai city, the provincial hospital has multidimensions as a provincial hospital for receiving referral cases from general hospitals, as the central general hospital for supporting other general hospitals and community hospitals, and as the community hospital for promoting community health care and health-promoting hospitals. The two general hospitals administer the upper and lower centers of the provincial area, while providing support to community hospitals under their hierarchical areas. The community hospitals provide health care services for people within the area of their amphoe and cooperate with the health-promoting hospitals or local healthcare units located in each amphoe.

Each district has a community hospital, while each community hospital, except for three, has at least one occupational therapist. The Chiang Mai province has the highest number of occupational therapists working in community hospitals in Thailand. Furthermore, one psychiatric public hospital located in this province provides mental health care for 17 provinces in northern Thailand, while the university hospital that is also located in this province provides mental health services for clients from Chiang Mai and the surrounding provinces as well.

This research context covers rural areas (long distance, over 60 kilometers or requiring more than an hour and 30 minutes to enter the area) and urban areas (less than 60 kilometers or needing an hour and 30 minutes to enter the serviced area). To explain the research context, this division is based on the distance or time spent to enter each community, starting from the Chiang Mai city hall. Further, all rural areas of OT service included in this study come from the southern parts. Entering the farthest service area typically takes half a day to reach the community, and community service providers must stay overnight. This context provided an example of how the landscape in rural areas influences the OT service. The sociocultural context in the urban area fuses modern and Lanna cultures, as reflected in the architectures, and lifestyles. Also, the people understand Thai well. The rural area has a similar culture but is less modern. The urban area is located in the plain along two sides of the Ping River. The landscape features in the rural areas are diverse with mountains, hills, alternating plains, and foothills as well as farms, while some parts of the rural areas in the plains and prairies are similar to the suburbs. The individuals living long distances from the Chiang Mai city wall and in high mountains speak a tribal language; they cannot understand Thai and believe in ghosts as part of their spiritual beliefs. Most of these residents are farmers and Buddhists.

## 3. Method

### 3.1. Research Design

We utilized a grounded theory methodology to understand and interpret social constructivism and pragmatism to construct a substantial theory [28] from the perspective and working concepts of occupational therapists. Social constructivism is “a theoretical perspective, which assumes that people create social reality or realities through individual and collective action” [29], while pragmatism views the realities of people as active and creative and looks the interplay between individuals in social groups and the worlds that they live [28, 29]. Pragmatism helps us to “see facts and values as linked rather than separate and truth as relativistic and provisional … through practical action to solve the problems…” [29]. The objective of grounded theory study is to generate a substantial theory and theoretical explanation in a particular area [28]. Consequently, the grounded theory methodology is appropriate for studying phenomena that lack detailed knowledge by developing new conceptual models directly from the data. This study employed the grounded theory methodology to understand, comprehend, and improve mental health care of COTS in the Thai context.

Symbolic interaction [29] was utilized to view language, action, and expression of a social relation between the participants and the patients, the participants and the patient's family, and the participants and other staff in the community or health care professionals. Theoretical sensitivity [30] was incorporated into our research because we upheld and recognized this concept as relevant to emerging theory. As Strauss and Corbin [30] stated, “theorizing is the act of constructing … an explanatory scheme that systematically integrates various concepts through statements of relationship.”

### 3.2. Ethical Considerations

The study was approved by the Tokyo Metropolitan University Research Ethics and Safety Committee (reference number of 19007, 2019) and the Human Research Ethical Committee, Suanprung Psychiatric Hospital (reference number 11/2562, 2019). All participants provided their informed consent before data collection. Pseudonyms were used to protect the identity of the participants in this research.

### 3.3. Reflexivity

A theoretical preunderstanding through a proactive approach [24] allowed the employment of a reflexive stance before and during the analysis. The researchers used written memos to document reflexive outlines of various viewpoints that could impact the analysis process. We mainly focused on the participants' clinical experience in community mental health practice, tackling sociocultural, policy, and political issues and the possibility of service in holistic care.

### 3.4. Recruitment

The research studied a group of occupational therapists. Two research participants were initially gathered using the initial sampling [29]. Inclusion criteria were (1) individuals working in a psychiatric hospital or community hospital in the Chiang Mai province Thailand and (2) individuals with at least one year of relevant experience in community mental health services for people with depression in Chiang Mai (calculated to the date of interviewing). Occupational therapists working with pediatric patients were excluded. Theoretical sampling [29] was employed for other participants. To provide an example of theoretical sampling, we initially grounded the setting and characteristics of the social participation support of COTS by interviewing two participants. However, following this, we had assumptions about the distance, teamwork, and service standpoint influencing social participation in COTS. Thus, theoretical sampling was used in the third participant, who provided service at farther distances, including rural areas. The participant was included to give more information for clarifying these assumptions, giving examples, and fulfilling the characteristics of rural area service, occupation, and cultural context of the patients in the area.

### 3.5. Data Collection

We used one to three face-to-face in-depth, semistructured interviews; nonparticipatory observations; memos; and field notes to collect data. An interview guide was developed to allow for flexible use and raise important issues within three areas, namely, (1) characteristics of social participation among patients with depression in the community mental health service, (2) factors related to social participation support for patients with depression, and (3) concepts and processes of developing social participation. Furthermore, the questions could be modified, and new questions were generated to investigate how an occupational therapist managed the service situations and proactive work factors. Each interview lasted 40–60 minutes and was audio recorded and transcribed. An observation guideline was developed to systematically document observations regarding the physical environment, activity atmosphere, attire, facial expression, the verbal and nonverbal language of the occupational therapists, and their interaction with the patients, the patients' family or community members, and other staff members while providing COTS (e.g., providing mental health knowledge and recreation activities, making home and community visits, organizing group activities, and promoting social skills and time management) in both hospital and community settings (e.g., community pavilions, village temples, patients' houses, and outpatient clinics).

We conducted 24 individual interviews, 14 observations, and one theoretical group interview from October 2019 to January 2020. The theoretical group interview [31, 32] was applied in the last data collection session to achieve data saturation. The theoretical group interview allowed us to find the “final missing pieces of the puzzle, polish data collection, and complete processes of saturation” [31]. To that end, the participants were contacted to determine their availability. Four participants participated in the theoretical group interview and agreed to have a group interview of 90 minutes per session with two sessions in a day. During the group interview, the researchers supplied brief preliminary findings, followed by asking the questions developed from the theoretical codes and constructs. Participants were then asked to explain their thoughts through examples and provide explanations of collaborative work within different institutional factors. Finally, the researchers identified an emerging model from the individual interviews. The researchers also encouraged all participants to give the information during the group interview to ensure that all perspectives were considered.

### 3.6. Data Analysis

Twenty-six transcripts, field notes, and memos were analyzed through the constant comparative method. We adopted Charmaz's analysis methods [29] by adding axial coding [30] as the link categories with subcategories and explaining its relation among events and occurrences [30]. Thus, initial coding, focused coding, axial coding, and theoretical coding were used in this research.

## 4. Results

This research included 14 participants (13 females and one male). All participants had work experience in uni- and multidisciplinary teams to care for patients with depression with and without comorbidities. Mean age was 37 years, with relevant experience ranging from three to 25 years (with a mean of 11 years). Five participants worked in regional hospitals, one in a university hospital, five in general hospitals, and three in community hospitals. Thirteen were affiliated with the Medical Services Department or the Mental Health Department in the Ministry of Public Health, while one was from the Department of Psychiatry, Chang Mai University.

**Figure 1:**
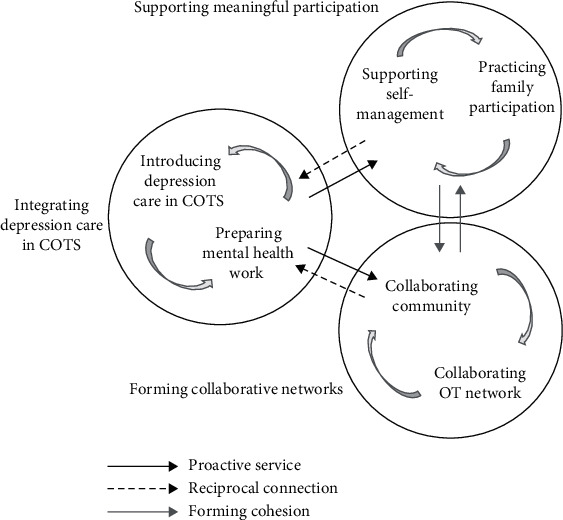
A proactive community occupational therapy service for depression (PCOTS-D) conceptual model for social participation development of Thai adults with depression.

The result identified proactive community occupational therapy service for depression (PCOTS-D) by illustrating three concepts of redeveloping social participation and reintegrating Thai adults with depression in their community. These three concepts were (1) integrating depression care in COTS, (2) supporting meaningful participation, and (3) forming collaborative networks. PCOTS-D supported the reconnection of social participation for integrating the three concepts. The participants started the process by searching for an opportunity to integrate depression care into COTS to work toward the other two concepts by assuming the roles of a supporter, coordinator, and facilitator. A reciprocal connection strengthening the first concept occurred by increasing awareness regarding depression care in the community and rebuilding positive social participation experiences among people in the community. The final goal of PCOTS-D was to form a strong community through collaboration between the patients and community members. Concurrently, the participants were able to attain professional development (Figure 1). In PCOTS-D, social participation is defined as an individual's ability to engage in daily activity and occupation with at least one person through interactive verbal and nonverbal communication.

### 4.1. Integrating Depression Care in COTS

This concept opened a new path for depression care of COTS in two categories: introducing depression care in COTS and preparing mental health work. The participants explored opportunities to integrate depression care by critically reviewing the service experience and suggesting improvement to COTS. A gap in COTS was found that rarely covered treatment for patients with depression, despite the increasing incidence of depression in both inpatients and outpatients with or without comorbidities. Furthermore, such services had weak community integration and mainly used a medical-based model in patients with a physical disability as a routine service in the hospital rather than a social model covering community-based service. These issues led to an imbalance in physical-mental-social health treatment and rehabilitation. Improving these issues would resolve a disjunction between the patient's intrapersonal and interpersonal relationships, especially in work and social participation, while decreasing the stigmatization of depression as a mental illness, which was a sensitive issue to address while working in the community.

“…Slow remission, conflict within families, and cause of despair and readmission are continuously found as hidden mental problems disturbing the lives of patients in their communities. Promoting community mental health services is needed in advocacy and combination with former community OT service, which mainly focuses on physical health.” (Chanisa, 33 years old, female)

The first category was *introducing depression care in COTS* toward a holistic intervention because “the promotion of mental health support could increase the quality of life and extended staying duration in the community” (Chanisa, 33 years old, female). The participants self-analyzed former COTS, while a few of them discussed this concept in a uniprofessional group in their units. The participants sought mental and social health support by applying PCOTS-D under the hospital's health promotion policy to deal with patient's common occupational problems, particularly poor emotional and time management, lack of leisure, job change or cessation, and social isolation. The participants initially introduced mental health in COTS by composing a checklist of OT services or presenting the role of the occupational therapist to physicians and nurses in the meeting. This introduction stimulated long-term rehabilitation, health promotion, and prevention in a community setting.

“Presentation of the OT role supports working in community mental health and holistic care under the health promotion policy. This decreases the patient's worry and despair even if they have or have not physical disability.” (Chalothorn, 42 years old, female)

Community-based rehabilitation (CBR) was the eventual goal of PCOTS-D. Concerning this, all participants raised awareness regarding sustainable mental health care for patients living in their community. As a participant, Chalitta (33 years old, female) said,

“I adapt the mental health service into the general COTS program to help individual(s) with depression. I integrate depression care in community mental health outreach. This provides specific mental health care through a multidisciplinary team and attempt using CBR for long-term care.”

The second category was *preparing mental health work*, including a workflow service and screening tool to enhance PCOTS-D, as the participants were responsible for reviewing and updating mental health knowledge. According to a participant, “preparation (for working in community occupational therapy service) seems a basic requirement, but it is a mandatory need in depression care and overall community mental health service” (Chanisa, 33 years old, female). The participants designed a service workflow for depression care, including social participation development into two service plans (individual and community as the second and third concepts, respectively). The workflow was organized and updated in the COTS plan for date and time, therapeutic activities, client group, responsible community staff, and issues of mental health knowledge needed in rehabilitation and health promotion. The participants employed this strategy for the outpatient ward (Muanjai clinic), inpatient ward (moderate to severe cases), community-based projects, and home visits.

“Mental health service requires a plan of serviced schedule, co-working staff, materials, and budget preparation more than regular work. I must work with sensitivity to any signs or symptoms and apply special techniques to individual clients following the limitations of community and hospital settings.” (Chutima, 29 years old, female)

A standardized screening tool should be developed for COTS to demonstrate the occupational problems directly and include the imperative social skill requirements. This screening tool should (1) recognize the importance of the patient's self-understanding and self-management and factors influencing social participation in their social life, (2) determine the individual-specific issues in social participation, and (3) assist in linking application occupational-based models such as the Person-Environment-Occupation-Performance Model (PEOP), the model of human occupation (MOHO), and the Canadian Model of Occupational Performance and Engagement (CMOP-E) to guide the practice. As a participant, Chanit (27 years old, female) said,

“I employed a 2Q-short form and 9Q questionnaires for screening depression. I need a tool that can show occupational and social problems that are important to help us determine OT-related problems and measure outcomes in depression. Currently, I use a non-standardized tool. A good (standardized) tool will support my work and serve as a reference in the multidisciplinary team.”

### 4.2. Supporting Meaningful Participation

This concept comprised two categories as a proactive approach to reconnect with oneself, one's family, and others in the community and workplace, while also inducing a sense of meaning and importance regarding social participation for health and recovery. The participants encouraged meaningful participation for the patients' self-management during family and community support; hence, the family was included as a part of the intervention process to promote the patients' full social participation.

The first category was *supporting self-management* for the patients' self-management. A participant said, “the patient can be likened to a dried tree with withered leaves and haulm, but the root may not be dead” (Chanapa, 35 years old, female). This assertion shows the participant's reflection in realizing a patient's performance and depression recovery to encourage and achieve self-management. The participants used a client-centered approach, contemplative education, and recovery model to identify the value of coexistence in the patients' social participation. For patients with moderate or severe depression, the participants assisted in exploring the patients' needs regarding occupation and activities of interest related to their social life instead of finding the aforementioned value. To support self-management, the patients were initially motivated by understanding and accepting that depression is a part of the present life that could be treated. The participants assisted the patients and their families to explore and express their feelings by recreating social attachments and highlighting the performance capacity in participation instead of focusing on social isolation. In this category, all participants suggested adapting the patient's preferred activities to maximize sensory perception, support decision-making during participation, and restrict depressive emotions with the experience of doing. Several activities (e.g., crafting, writing a diary, and doing macramé) were adopted in this process to support psychological flexibility through emotional and behavioral adaptations. Participants allowed the patients to take as much time as needed to either personally accept their circumstances or understand the purpose of self-exploration. The family members of the patients were the primary supportive unit in the gradual change from sadness and complaining about themselves to realizing and accepting self-management. Other activities such as time management, pet therapy or pet ownership, group activities (games or social skill training sessions), and activities designed using an adaptive Snoezelen room were suggested to promote self-exploration and management and manage the mood in choosing leisure activities. As a participant, Chalothorn (42 years old, female) said,

“I help the patients understand and manage their lives through daily activities. They are overwhelmed with negative ideas about themselves and others and lose connection. It is crucial to ensure that they know the meaning behind their thoughts.”

The second category was *practicing family participation*. Families of the patients were educated about depression symptoms as influencing factors and the benefits of social participation and occupational engagement. All participants stated that the patients believed that their families' health is equivalent to their own social and mental health. The participants provided tips to a family member, specifically listening and practicing *sati* (mindfulness), to create a therapeutic relationship between that family member and the patient. This was the first step in introducing social support from the family and building trust internally in the family before encouraging patients to spend more time with their families when living at home or in the community. Mealtimes, gardening, having a picnic, praying, shopping, playing games, exercising, going to the gym, joining clubs, religious activities, and visiting festivals were identified as the therapeutic activities of social participation in the home and community environments. Moreover, the participants used the same method for hospitalization and discharge preparation. As a participant, Chanapa (35 years old, female) said,

“Family participation is the first step before returning to other social or work activities because Thai adults have family or loved ones within their life-value and happiness. Family participation provides an opportunity to recover family trust as the best social support for Thais. Thus, optimal and sufficient family participation is needed to regain the patient's social experience and inside recovery by having their family and myself as outside supporters.”

### 4.3. Forming Collaborative Networks

This concept illustrated two categories, namely, forming a community network for depression care and coworking with an OT network. A participant emphasized, “promoting social participation in the patient during COTS involves working between the therapist and the patient and also with the community” (Chayanit, 43 years old, female).

*Collaborating with the community* as the first category promoted three purposes. First, the participants highlighted that, by gaining knowledge of depression, people in the community can have a more positive attitude regarding depression and reduce its stigmatization and discrimination. This often negatively affects individuals' confidence and self-worth, causes poor self-management during work, and leads to social participation problems, social deprivation, and suicidal ideation. Second, by supporting patients' work or employment, their performance in work and community participation, including extended duration of living in the community, can be restored. The participants emphasized the importance of the patients' social structure (community and workplace) and promoted collaborating with the community network to strengthen it. The participants adapted each community's knowledge and resource and crafting famous products into vocational rehabilitation by starting projects such as growing vegetables and rearing livestock, making batik and cloth dyes, and practicing arts and crafts. Successful work promotion in the community was observed in highly collaborative communities. Third, by creating a supportive physical and natural environment within the community, the participants encouraged the patients and community staff to use natural settings and community facilities such as parks, yards, gardens with foliage, fountain, hot springs, and Thai massage shops to support good mental health and promote engagement with other people in the community (e.g., pet ownership, exercise, and spending leisure and family time in public places).

For such collaboration, the participants worked with healthcare staff from health-promoting hospitals (psychiatric nurses, public health technical officers, and health volunteers) and the local government (municipality or subdistrict administrative organization). The local government and staff were crucial in managing and redeveloping social interaction and participatory relationships between patients and patients, patients and staff, and patients and family members and neighbors. The participants, along with several community members as knowledge providers, trained the village health volunteers to engage in knowledge distribution and mental health promotion. Thus, this collaborative work induced a sense of belonging between the patient and community members. As a participant, Chollana (33 years old, female) said,

“I value working with health-promoting hospitals and local government. This community team is vital for long-term development and support to promote patients' community participation. They support work that the patients value. The products made can be sold all year and relate to northern culture. The patients work with other people and return from their world of isolation to recover their self-worth. Collaborating with the community has both direct and indirect benefits to the patients.”

*Collaborating with an OT network* was the second category to develop a supportive network and knowledge evidence. Some participants who worked in community hospitals coordinated with senior occupational therapists as mentors from the area's center to boost their confidence and prevent burnout. The participants also collaborated with OT researchers in academic institutions to conduct research and generate knowledge evidence. This collaboration motivated them to seek promotion at their workplace and gain hands-on experience in local government policies. The participants reflected on collaborative work, appreciated the value of COTS in mental health, and challenged proactive work in the community context rather than merely engaging in routine work at the hospital. All participants agreed to conduct research that increased social impact and had high potential to advocate for supportive policies. Collaborating with researchers provided the opportunity to coordinate among different hospitals and institutional policies that cover depression care. As a participant, Chayanit (41 years old, female) said,

“We (occupational therapists) work to address mental problems and also social problems in people suffering from depression. We need to increase awareness through effective communication and publication. Social support can decrease stigmatization, while publications create evidence to understand how best to regain participation and socialization. Our profession has a limited workforce; thus, we need collaboration.”

## 5. Discussion

The research results illustrated that the PCOTS-D conceptual model identified three main concepts as integrating depression care in COTS, supporting meaningful participation, and forming collaborative networks as working processes of occupational therapists in social participation redevelopment for community-dwelling Thai adults with depression. PCOTS-D introduces a new perspective of COTS that fits Chiang Mai's community mental health care context in both rural and urban areas; further, this mental health challenge has persisted in the changing health care paradigm worldwide from 20 years ago to date [33–37].

The proactive approach supports the occupational therapist's role in the reform of mental health services from a hospital or institution to a community [34] amid several challenges impacting the opportunity of improving the personality characteristics of the therapist in mental health care. The participants would be trained to be sensitive to the signs and symptoms of depression [38, 39] and also to be calm and mindful listeners to address the need to respond to the patients' unpleasant emotions [40]. These characteristics encourage their supportive role in stimulating the patients' prominent need for self-exploration while they simultaneously facilitate the process through which the patients' families support them to be ready for reparticipation. Many studies revealed that the patients' feeling of readiness is critical because consciousness is a basic need for raising their inward motivation for participation while receiving compassionate outward support [41–44]. Another challenge working on PCOTS-D is providing interdependent [41], mindful [41], and contemplative education [40] to benefit emotional observation, behavioral management, and self-development associated with Thai social culture.

On the other hand, all participants perceived several barriers to the professional development of PCOTS-D, such as funding, setting, mental health knowledge, clinical workload, time limitation, and expectation of tangible outcome within a short duration [45, 46] as well as sensitive issues of mental health service (social attitude toward home visits or use of the word “depression” resulting in stigmatization) [47–49]. This issue concerns an ethical dilemma regarding the clinical interventions for mental disabilities [50] because the participants advocated for the right to depression rehabilitation and the opportunity to express the patients' need for social support; the existing negative viewpoint could be seen as the threat to working with COTS, such as resorting to secret or underhanded means or infringing on one's privacy. The lack of knowledge regarding the occupational therapist's role forces the participants to deal with the surrounding social attitudes. Further, another social attitude is the perception that depression is motivated by attention seeking [51] rather than support seeking. Again, this attitude enhances the threat of social deprivation and discrimination, while the participants must develop conciliatory communication skills in working with ethical dilemmas to encourage accessibility of mental health rehabilitation in the community while decreasing stigmatization [52, 53].

As the research result, the participants realized the patient's capability for self-determination and recovery. This realization is an essential facet of mental health recovery related to the meaning of life, responsibility, hope, and self-identity [16]. It helps to empower the patients to assume ownership and maintain an active role in their recovery process and social connection [54]. Likewise, the participants enhance the patient's capacity for self-awareness and self-management in emotional and behavioral adaptations similar to the process of recovering from depression in the Thai situation studied by Chanapan [55], which discovered that understanding attitudes, values, feelings, goals, behaviors, skills, and roles is necessary for inner self-reconnection. This study learned and adapted from the changes recommended by Chanapan. Thus, in supporting the patient's performance, occupational therapists must employ a client-centered approach along the journey of personal recovery through self-management to motivate them to engage in meaningful activities, participate, and attain their requisite outcomes.

Promoting self-management, especially among those from the rural areas, attached more value to the people around them rather than money, although the patients' value and motivation were previously tied with work and money due to insufficient income [56, 57]. Moreover, linking value to the surrounding people leads to a positive attitude toward living a simple life. This illustrates happiness [58] by depicting a change of values from making a lot of money to a basic living within the community and self-determination through occupations such as agricultural work, according to the Sufficiency Economy projects of His Majesty King Rama 9 to promote living with economic sufficiency in the rural area. Utilizing hospital policy support, a participant created a project to promote the patient's need for work and social participation within each work process and reconnected them to the community. The community workers also understood the patient's working capacity. This helped decrease stigmatization and increase self-management for occupational performance recovery. This project was applied to all people with disabilities in that area, including persons with depression. This example project distinctly tackled the patients' value and motivation changes and facilitated community support to redevelop social participation with real meaning behind the patients' expression and participation.

Challenging the notion of protecting their family from experiencing *lao-kwuan* (other people talking badly or blaming the family for having an ill person in the household) was one of the requirements of patients' self-management. This is similar to gossiping, resulting in stigmatization [48, 59]. This notion can impact the patients' motivation, causing either social isolation or engagement in hard work to show functional performance. Hence, there is a need to transform this unhealthy notion through a reorganization process that will increase resilience through self-management [60]. Future research can be undertaken to determine what activity or intervention program is suitable for the promotion of resilience in the redevelopment of social participation and enhancement of optimal participation in individuals' everyday life. This will assist in helping the individuals to learn the meaning of participation [54] and promote their trust, which is the first key to the readiness of patients' participation.

Lastly, the collaborative network development of PCOTS-D produces sustained connections in the patient–family–community–profession collaboration. It results in building a strong community that benefits not only depression care in long run but also mental health for all, which is the new path of mental health care of COTS in the Thai context. The community network system is a valuable resource [49, 61, 62] and impacts proactive work because it serves as a crucial strategy of accessibility and sustainability in Thailand's mental health programs [61]. The participants endeavored to follow this observed trend and found that only communities with a strong sense of collaboration experienced the successful development of family and community participation. In the proactive approach, the collaborative network expands from an interprofessional team in the hospital to the community authority because the community becomes the new rehabilitation place for the patients. A strong collaborative network would most likely form because most neighbors within the Thai community and society are relatives or friends. Furthermore, engaging in work within the community network provides opportunities to receive support from the economic and local facilities under the national universal insurance coverage [61]. For example, community policy has a massive influence on developing a physical and natural environment that supports mental health promotion, for instance, green and relaxing environments and numerous social assets such as volunteers [63]. Network development should be supported and encouraged to counteract the lack of Thai occupational therapists [26]. However, the hospital and community policies are the institutional environment's variables. Hospital policies are the most significant factors for the participants working toward the development of a supporting network. This issue is related to the infrastructure of the public health service system of the Ministry of Public Health [61].

Additionally, the collaboration with an OT network is crucial for advocating for a supportive policy related to knowledge management and transference, which is the focus of the Bureau of Mental Health Technical Development [61]. The strong network of occupational therapists for COTS comprises the Chiang Mai Occupational Therapist Club, senior occupational therapists at the mental health hospital, the university and general hospitals, and researchers in an OT school located in the context. This professional empowerment strengthens the collaborative network in this context, enhances sharing and exchange of comprehensive knowledge such as the recovery model in practice and applied contemplative education for service improvement, and promotes engagement in knowledge management and research development [27]. Thus, forming a professional collaborative network should be highlighted as a solution to today's social situation [54]. Doing so will ensure effective and up-to-date COTS and reduced risk for burnout in the occupational therapists [36]. Finally, the second version of the mental health legislation was launched in the past few years [27]. Invoking the policies within the said legislation, PCOTS-D can grant rehabilitation for depression or health promotion through collaborative networks. However, its support for substantial rehabilitation and health promotion programs is less demonstrated. Thus, occupational therapists must garner support from various alliance organizations and advocate for increasing the OT workforce delivering community services for the care of depression.

### 5.1. Strengths and Limitations

This research conducted an in-depth exploration of the subject matter through a grounded theory approach using a constant comparative analysis based on comparing and contrasting data among authors. Triangulation was employed to confirm the research results with the participants' experiences [64]. This research was grounded in Thai social contexts and had the credibility to ensure enough data merit, achieving familiarity with the Chiang Mai context. Three authors' joint discussions demonstrated reflection during data collection and revealed real meaning, which resonated with a true sense of participants' experience and circumstances. A typical limitation of qualitative research is its ability to generalize results outside similar OT service contexts in upper northern Thailand. Should other studies in different contexts plan to utilize the result of this study, the sociocultural context of this context should be reflected upon and contrasted with that of the proposed study.

## 6. Conclusions

The research presented PCOTS-D as three concepts to redevelop social participation for community-dwelling Thai adults with depression from the perspective of occupational therapists. PCOTS-D supports a holistic view of working with patients with depression by understanding and intervening in the patient's community and with OT networks. PCOTS-D presented the redevelopment of social participation from an individual context of participation in promoting health and recovery and subsequently, the immediate context, through forming a collaborative network with community staff and other occupational therapists for all dimensions of depression care. Lastly, the development of social participation is more than helping to restore a patient's potential or managing emotional and behavioral expression. Family, community, and social collaboration must be harnessed to resolve the real-life social participation problems sustainably. Thus, these research results fulfill the local government's emphasis on valuing its community member's health care and supporting community resources, leading to a strong and independent community of Thai people.

## Data Availability

All necessary information that supports the findings of this study is provided within this manuscript. Raw data would remain confidential and would not be shared because of ethical concerns.
